# Dynamic and Differential Regulation of Stem Cell Factor FoxD3 in the Neural Crest Is Encrypted in the Genome

**DOI:** 10.1371/journal.pgen.1003142

**Published:** 2012-12-20

**Authors:** Marcos S. Simões-Costa, Sonja J. McKeown, Joanne Tan-Cabugao, Tatjana Sauka-Spengler, Marianne E. Bronner

**Affiliations:** 1 Division of Biology, California Institute of Technology, Pasadena, California, United States of America; 2 The Weatherall Institute of Molecular Medicine, University of Oxford, Oxford, United Kingdom; Osaka University, Japan

## Abstract

The critical stem cell transcription factor FoxD3 is expressed by the premigratory and migrating neural crest, an embryonic stem cell population that forms diverse derivatives. Despite its important role in development and stem cell biology, little is known about what mediates FoxD3 activity in these cells. We have uncovered two FoxD3 enhancers, NC1 and NC2, that drive reporter expression in spatially and temporally distinct manners. Whereas NC1 activity recapitulates initial FoxD3 expression in the cranial neural crest, NC2 activity recapitulates initial FoxD3 expression at vagal/trunk levels while appearing only later in migrating cranial crest. Detailed mutational analysis, in vivo chromatin immunoprecipitation, and morpholino knock-downs reveal that transcription factors Pax7 and Msx1/2 cooperate with the neural crest specifier gene, Ets1, to bind to the cranial NC1 regulatory element. However, at vagal/trunk levels, they function together with the neural plate border gene, Zic1, which directly binds to the NC2 enhancer. These results reveal dynamic and differential regulation of FoxD3 in distinct neural crest subpopulations, suggesting that heterogeneity is encrypted at the regulatory level. Isolation of neural crest enhancers not only allows establishment of direct regulatory connections underlying neural crest formation, but also provides valuable tools for tissue specific manipulation and investigation of neural crest cell identity in amniotes.

## Author Summary

FoxD3 is an important stem cell factor expressed in many types of embryonic cells including neural crest cells. In the embryo, neural crest cells are a type of stem cell that forms diverse derivatives, including nerve cells, pigment cells, and facial structures. To better understand neural crest development and differentiation, we have explored how FoxD3 expression is regulated in these cells. By examining non-coding DNA, we have identified distinct genomic regions that mediate expression of green fluorescent protein (GFP) in a pattern that recapitulates FoxD3 expression. Interestingly, we find two genomic “on–off” switches or enhancers, called NC1 and NC2, that drive GFP expression in a pattern that recapitulates FoxD3 expression at different times and places during neural crest development. We find that Pax and Msx proteins turn on both NC1 and NC2 enhancers by directly binding to them. In addition, cranial expression driven by NC1 requires a protein called Ets1, whereas trunk expression of NC2 requires a different protein called Zic1. The results show that FoxD3 in differentially regulated in distinct neural crest cell populations in a manner that is specifically encoded in the genome. These enhancers provide valuable tools for understanding neural crest development in birds and mammals.

## Introduction

The neural crest (NC) is a transient population of cells that migrates throughout the embryo and forms many different cell types, including neurons and glia of the peripheral and enteric nervous systems, bone and cartilage of the craniofacial skeleton and melanocytes [1], [2]. Induction of the neural crest is thought to involve a number of growth factors, including Wnts and BMPs, that establish the neural plate border region that contains the prospective neural crest. This region is characterized by the collective expression of a number of transcription factors, including Msx1/2, Pax3/7 and Zic1, termed neural plate border genes [3]. Subsequently, as neurulation progresses, additional transcription factors are expressed by neural crest precursors residing within the neural folds and dorsal neural tube. These transcription factors, termed neural crest specifier genes, include Sox9, FoxD3, Ets1, Snail1/2 and Sox10, amongst others [2]. Regulatory interactions between neural plate border genes, neural crest specifier genes and signaling inputs generate a complex gene regulatory network (GRN) that orchestrates essential steps in neural crest ontogeny, including emigration from the neural tube, migration to appropriate locations and differentiation into many different cell types.

An important challenge is to establish direct connections within the neural crest GRN. For example, the neural plate border marker, Pax7, is essential for expression of a number of different neural crest specifier genes [4] such that its loss-of-function results in the subsequent loss of Sox10 and Snail2 in the cranial neural crest. Thus, these genes act downstream of Pax7, either by direct or indirect interactions. For the case of Sox10, regulatory analysis revealed direct inputs from Sox9, Ets1 and Myb, but not Pax7 [5], suggesting that effects of loss of Pax7 on Sox10 expression are likely to be indirect. This raised the question of what genes might be direct targets of neural plate border genes like Pax7.

Of the neural crest specifier genes, FoxD3 is one of the first markers of premigratory neural crest in many vertebrate species including mouse, chick, *Xenopus* and zebrafish [6]–[12]. Its initial expression in the neural tube precedes that of Sox10 and several pieces of evidence suggest that FoxD3 is critical for initiating a cascade of neural crest gene expression that controls their emigration from the neural tube. For example, ectopic expression of FoxD3 in the chick neural tube induces expression of neural crest markers and increases emigration from the neural tube [13]. Similarly in *Xenopus* ectopic expression at the 8-cell stage increases the expression of neural crest markers, while expression of dominant-negative FoxD3 reduces expression of genes like Snail2, Twist and Ets1 [11] and depletes some neural crest derivatives [9], [11], [14], [15].

Despite its important role both in stem and neural crest cells, no regulatory element(s) controlling the onset of FoxD3 expression are known. To define linkages and assess direct regulatory interactions in the neural crest gene regulatory network with particular interest in possible targets of Pax7, we set out to dissect the *cis*-regulatory regions of the critical neural crest gene, FoxD3. Taking advantage of the chick's compact genome and ability to assay putative regulatory regions by electroporation, we have identified two enhancers, NC1 and NC2, that mediate reporter expression in spatially and temporally distinct manners in the chick embryo, and in combination closely recapitulate the endogenous expression of FoxD3. Detailed regulatory analysis shows that initial expression of FoxD3 in both cranial and trunk neural crest requires direct input from neural plate border genes, Pax7 and Msx1/2. These factors function in combination with the neural crest specifier gene, Ets1, to bind to the cranial NC1 regulatory element. However, at vagal/trunk levels, they function together with the neural plate border gene Zic1 to activate the NC2 enhancer. These results not only reveal region-specific enhancer activity in the neural crest, but also expand the neural crest GRN and inform upon direct interactions therein. Conserved between mouse and chick, these enhancers further provide excellent tools for assaying gene regulation and manipulation of neural crest gene expression in amniotes.

## Results

### Endogenous pattern of chick FoxD3 expression in the neural crest

**Figure 1:**
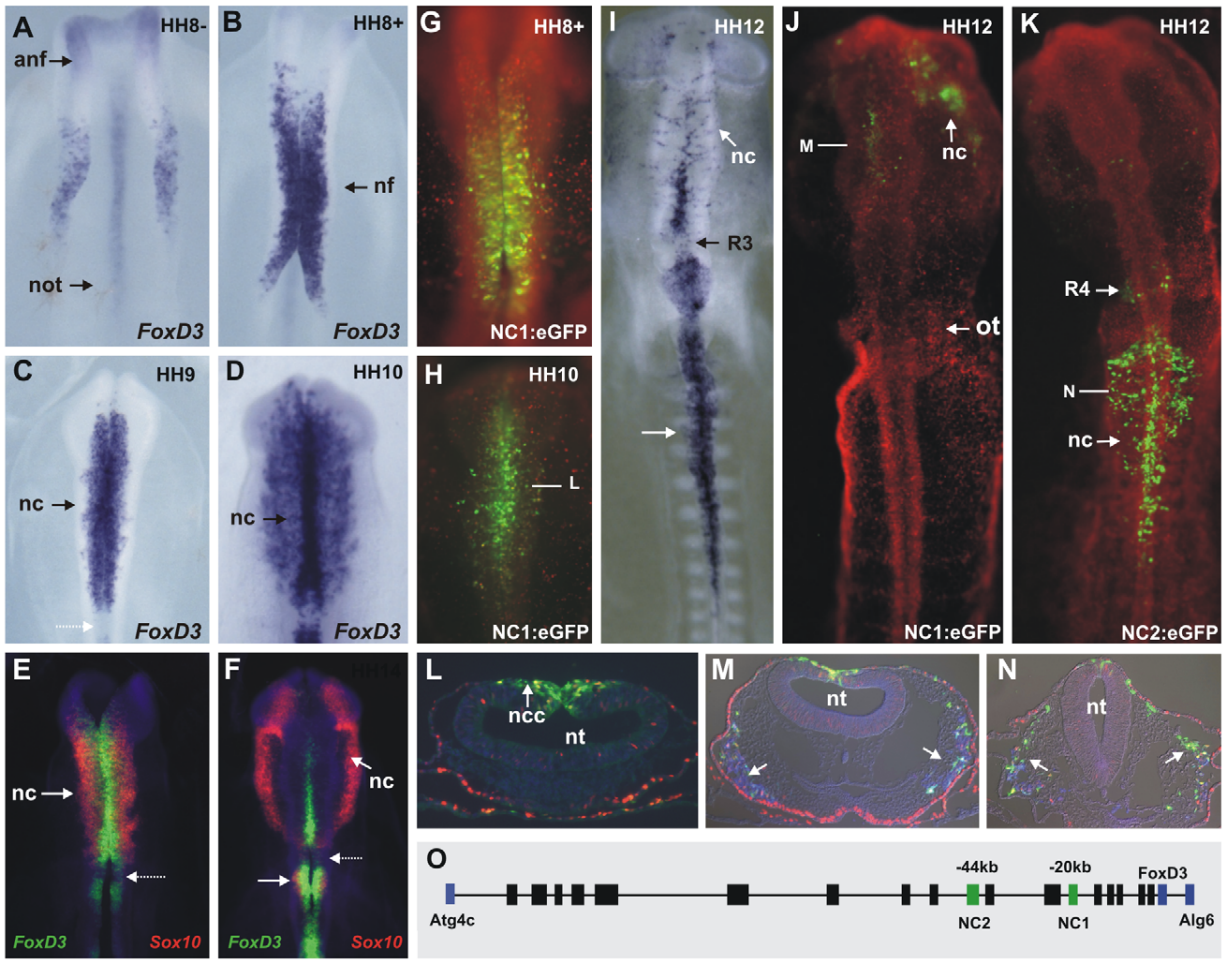
Endogenous FoxD3 in the neural crest is reflected by activity of two enhancers, NC1 and NC2. (A) Expression of FoxD3 in premigratory neural crest cells at HH8−. (B) At HH8+, FoxD3 expression extends to the midbrain and hindbrain neural folds. (C) At HH9, FoxD3 is expressed by premigratory and migrating neural crest cells, at cranial, vagal and trunk levels with the exception of rhombomere 3 (dotted arrow). (D) FoxD3 transcripts are detected in migrating cranial crest cells at stage HH10. (E–F) Double fluorescent *in situ* hybridization for Sox10 (red) and FoxD3 (green) reveals differences in the expression domains of these neural crest specifiers at stages HH9 (E) and HH10 (F). Expression of Sox10 begins only as cells leave the neural tube at all axial levels. (G–H) Expression of eGFP driven by enhancer NC1 at stages HH8+ and HH10. Bar indicates approximate level of transverse section shown in L. (I) *In situ* hybridization of FoxD3 at stage HH12. (J) Expression of eGFP driven by enhancer NC1. (K) eGFP driven by enhancer NC2. (L–N) Transverse sections through embryos shown in H, J, K. Arrows indicate migratory neural crest expressing eGFP. HNK-1-positive cells shown are in blue in M and N. (O) Genomic region of FoxD3 in chick showing regions tested for enhancer activity between the flanking genes Atg4c and Alg6 (blue boxes). Boxes indicate regions that were tested for enhancer activity: black boxes indicate no detectable activity in the neural crest; green boxes indicate enhancers active in the neural crest. Coding regions are indicated by blue boxes. anf: anterior neural fold, not: notochord, nf: neural fold, nc: neural crest, ncc: neural crest cells, nt: neural tube, ot: otic placode, R: rhombomere.

In the cranial neural tube, expression of FoxD3 initiates in premigratory neural crest cells at HH8- (Figure 1A), with strong and rapid onset of expression that precedes that of Sox10 or Snail2. At this stage, the *FoxD3* expression domain includes the neural folds of the forebrain and midbrain. Subsequently, at HH8+, FoxD3 expression expands posteriorly to the hindbrain (Figure 1B). As neural crest cells delaminate at HH9 and migrate at stage 10, FoxD3 is maintained or expressed *de novo* at high levels by many migrating cranial crest cells (Figure 1C, 1D). The domain of expression of FoxD3 at this stage includes premigratory and migratory crest extending from the midbrain to the trunk, with the exception of the neural folds at the level of rhombomere 3 (dotted arrow in Figure 1C). Expression persists through subsequent stages as the neural crest advances to surround the optic vesicle and populate the first branchial arch.

At vagal and trunk levels, FoxD3 is also expressed by premigratory and migrating neural crest cells. FoxD3 transcript expression initiates as the neural folds appose in the midline at stage HH9. At HH12, FoxD3 is detected in migrating vagal crest as the first wave of cells leaves the neural tube (white arrow in Figure 1I) and with time, FoxD3 expression initiates at progressively more caudal levels in the trunk neural tube and migrating neural crest. At later stages, FoxD3 expression is maintained in a subset of neural crest derivatives, including peripheral ganglia [16].

Double fluorescent *in situ* hybridization for FoxD3 and Sox10 reveals differences in the expression domains of these neural crest specifier genes. *FoxD3* transcripts are detected in the premigratory population prior to Sox10, which is expressed in cranial, vagal and trunk neural crest cells only as cells leave the neural tube (arrows on Figure 1E and 1F). Therefore, even though FoxD3 and Sox10 both have been placed in the same hierarchical level in the neural crest GRN, they are recruited at different time points during neural crest specification.

### Dissection of the FoxD3 regulatory region

The genomic region of FoxD3 was examined for conservation across multiple vertebrate taxa including chick, mouse, human, opossum, *Xenopus* and zebrafish using the UCSC Genome Browser and ECR Browser. The region analyzed spanned 160 kb between the genes immediately up and downstream of FoxD3, Atg4C and Alg6 respectively (Figure 1O). To test putative enhancers for neural crest regulatory activity, eighteen conserved regions varying in size from 1 kb to 4 kb were cloned into an eGFP reporter vector [17] and electroporated into the entire epiblast of stage HH4 or dorsal neural tube of HH8–14 chick embryos, together with pCI-H2B-RFP as a ubiquitous tracer to verify efficacy of transfection.

### Two enhancers, NC1 and NC2, mediate different spatial and temporal expression patterns in the neural crest

By testing conserved regions within the FoxD3 locus, we found two enhancers that drive specific expression of eGFP in the neural crest, in a manner that collectively closely recapitulates the endogenous pattern of FoxD3 expression. Enhancer NC1 directed expression of eGFP in the premigratory cranial neural crest analogous to the early endogenous expression of FoxD3 (Figure 1G–1H). eGFP in the cranial neural folds was detected from stage HH8+ (Figure 1G), in the dorsal neural tube and on a few neural crest cells during emigration (Figure 1H, 1J, 1L), lasting until approximately stage HH14, at which time only very weak eGFP expression could be detected. While migrating neural crest from the midbrain to rhombomere (R) 2 exhibited NC1 mediated eGFP expression (Figure 1J), no eGFP expression was observed caudal to R3. This contrasts with the endogenous FoxD3 pattern, which is observed in R4, R6 and more posterior crest (Figure 1I).

Enhancer NC2, in contrast to NC1, mediated strong eGFP expression in the premigratory, delaminating and migrating neural crest at and caudal to R6 (Figure 1K), beginning at HH9. This expression pattern of eGFP in the vagal and trunk neural crest recapitulates endogenous expression of FoxD3 (Figure 1I) at this axial level. The expression of both eGFP and endogenous FoxD3 mRNA extends to the premigratory/delaminating crest at the level of the 4^th^ most caudal somite. Interestingly NC2 activity also controlled eGFP expression in a large subpopulation of migrating cranial neural crest at the level of the midbrain, R1 and R2, which was detectable only after stage HH9+, and expression in premigratory and migratory NC from R4.

To examine the activity of NC2 enhancer at later stages and in neural crest derivatives, we electroporated stage HH8–14 embryos *in ovo*, and fixed the embryos after 24–48 h (HH15–20). FoxD3 is expressed in most premigratory and migratory vagal and trunk neural crest, but is down-regulated in melanoblasts (Figure S1A), which migrate underneath the ectoderm and initiate emigration approximately 24 h after the emigration of ganglionic neural crest in the chick. Interestingly, we observed expression of eGFP in melanoblasts prior to and during migration (Figure S1B), in addition to expression in the dorsal root and trigeminal ganglia. To confirm that this expression was due to activity of the enhancer and not stability of eGFP, we performed *in situ* hybridization for eGFP and detected mRNA for eGFP in melanoblasts and dorsal root ganglia (Figure S1D–S1E), suggesting that eGFP is indeed ectopically expressed by melanoblasts, under control of the NC2 enhancer. Expression of eGFP was also seen in neural crest cells migrating along the enteric neural crest pathway (Figure S1F). In contrast to NC2, at HH14 very weak expression of NC1 activity was confined to the branchial arches whereas no expression was observed in cranial ganglia.

**Figure 2:**
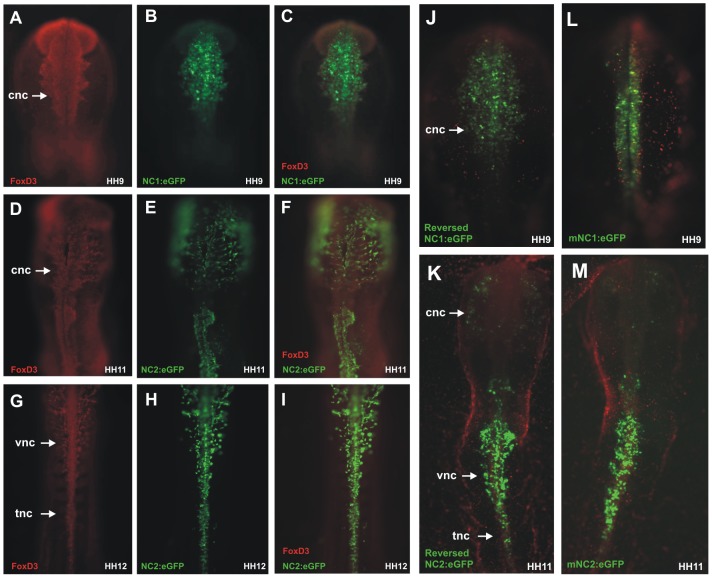
Chick NC1 and NC2 enhancers drive expression that overlaps with endogenous FoxD3 expression, function in reverse orientation, and are conserved with mouse NC1 and NC2. (A–C) Immunostaining with anti-FoxD3 antibody (red) of embryos electroporated with NC1:eGFP (green) shows overlap of enhancer activity and endogenous expression of FoxD3 in early cranial neural crest cells. (D–F) Migrating cranial neural crest cells express FoxD3 and eGFP driven by the NC2 enhancer in stage HH11 embryos. (G–I) Vagal and trunk neural crest cells that are positive for FoxD3 also express eGFP driven by the FoxD3 enhancer. (J–K) Reversing the orientation of NC1 and NC2 does not alter their ability to drive eGFP expression. (L–M) Genomic regions homologous to the NC1 and NC2 enhancers cloned from mouse (mNC1 and mNC2) drive expression of eGFP in a manner identical to chick NC1 and NC2. cnc: cranial neural crest, vnc: vagal neural crest, tnc: trunk neural crest.

We next examined overlap of endogenous FoxD3 expression with reporter expression driven by NC1 and NC2 by performing double labeling with eGFP and FoxD3 antibodies. The results show that NC1-driven eGFP expression completely overlapped with that of endogenous FoxD3 protein in stage HH9 embryos (Figure 2A–2C). Similarly, NC2-driven eGFP expression in migrating cranial neural crest overlapped with endogenous FoxD3 protein expression at stage 11 (Figure 2D–2F) and with FoxD3 in delaminating and migrating crest at the trunk/vagal levels at stage 12 (Figure 2G–2H). The complete overlap between enhancer activity and FoxD3 expression strongly suggests that NC1 and NC2 are the responsible regulatory modules for the control of endogenous FoxD3 in the neural crest.

To determine if orientation of the enhancers affects their activity, NC1 and NC2 enhancers were cloned in reverse orientation into ptk-eGFP and electroporated in HH4 embryos. The results show that both have equivalent ability to drive reporter expression in reversed as in their endogenous orientation, without significant changes in pattern or levels of activity (Figure 2J, 2K).

Finally, we examined whether these enhancers were conserved across amniotes. To this end, we cloned the homologous conserved regions from the mouse genome and mouse (m) NC1 and mNC2 constructs were electroporated into chick embryos at gastrula stages. The results show that the patterns of eGFP expression driven by mNC1 and mNC2 were identical to those observed with chick NC1 and NC2 (Figure 2L, 2M), suggesting that these enhancers are conserved between chick and mouse and likely throughout amniotes.

### Dynamic analysis of NC1 and NC2 reporter expression

To examine the dynamic nature and combined activity of the two enhancers in the migrating cranial neural crest, we co-electroporated NC1 (green) and NC2 (blue) enhancers in combination with a previously identified cranial neural crest Sox10E element (red) [5] that expresses in all emigrating and migrating neural crest cells. Reporter expression of multiple fluorophores was then visualized in transverse sections of slices through the midbrain region, using a novel slice culture protocol [18].

**Figure 3:**
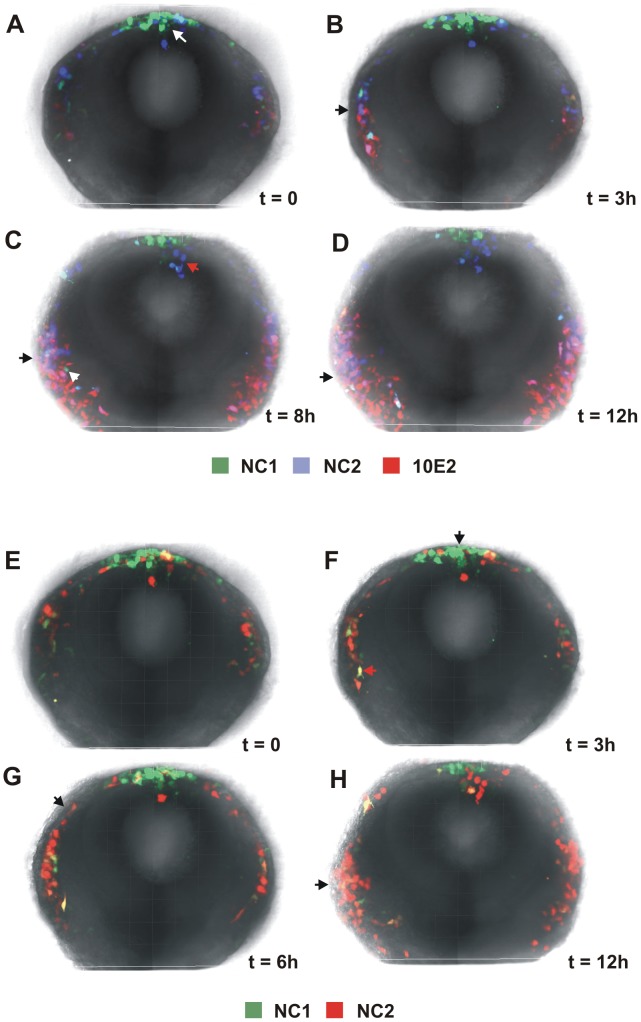
Dynamic regulation of FoxD3 and Sox10 enhancers in the cranial neural crest. (A–D) Selected images from a time lapse sequence showing activity of the NC1 (green) and NC2 (blue) FoxD3 enhancers and the Sox10E2 (red) enhancer in a chick cranial slice preparation (see Video S1). (E–H) Images from a time lapse movie of migrating cranial neural crest cells electroporated with NC1:eGFP and NC2:Cherry (see Video S2).

Time-lapse movies revealed differential temporal and spatial activity of NC1 and NC2 enhancers. While NC1 activity was present in the premigratory neural crest, the expression it drove in the dorsal neural tube appeared transient in most cells and preceded that driven by the Sox10E enhancer (white arrow in Figure 3A). NC1 activity then recurred in a small subpopulation of actively migrating cranial crest cells that concomitantly displayed Sox10E activity (black arrows in Figure 3B and 3C, Video S1). In contrast, NC2 activity was observed in very few cells within the neural tube (red arrow in Figure 3C), and only a few delaminating neural crest cells coincident with Sox10E activation. Thereafter, NC2 drove expression in a large subset of migrating cranial neural crest cells, which were also positive for Sox10E activity (black arrows in Figure 3C and 3D).

To further investigate neural crest heterogeneity with respect to enhancer-driven expression, we co-electroporated embryos with NC1 (green) and NC2 (red) enhancers and observed neural crest formation and migration by time lapse microscopy. Analysis of the movies suggested that there was little overlap between cells showing activity of NC1 and those with NC2 (Figure 3E–3H, Video S2). Only a few cells co-expressed eGFP and RFP and this may reflect perdurance of the reporter that may be more stable than the endogenous transcription factor. These results suggest that there is highly dynamic regulation of FoxD3 in migrating neural crest cells and suggest that there may be distinct subpopulations that reflect activity of NC2 but not NC1, or vice versa. They further suggest that NC1 activity may be largely responsible for the transient early expression of FoxD3 in the neural tube (arrow in Figure 3F), whereas NC2 activity at cranial levels may be primarily responsible for FoxD3 expression in migrating neural crest cells (arrows in Figure 3G and 3H).

### A conserved 80 bp element is the minimal essential core regulatory element of enhancer NC1

**Figure 4:**
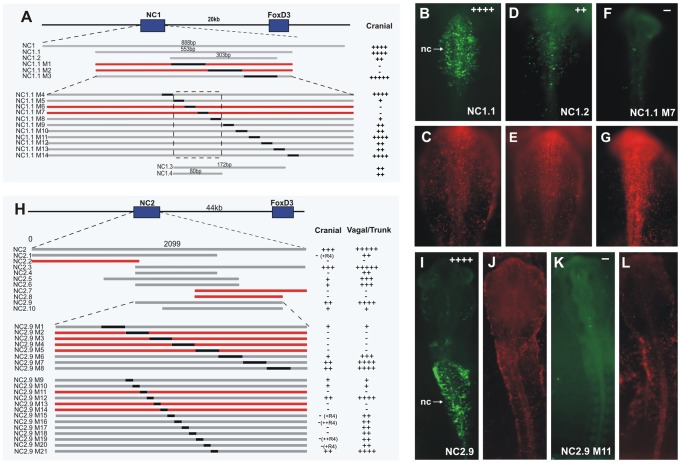
Dissection of the NC1 and NC2 enhancers. (A, H) Diagram of deletions and substitutions made to uncover critical enhancer regions. Each numbered bar represents a region of the enhancer that was tested via whole embryo electroporation. The relative level of expression in cranial and vagal/trunk neural crest (NC) is indicated on the right for each region ranging from + indicating weak expression to +++++ indicating strong expression. Gray bars indicate those enhancer regions that drove activity in the neural crest. Red bars indicate a lack of activity. Black fragments of the enhancer indicate substitution with GFP coding sequence. NC1.3 and NC1.4 contain the core region of the enhancer NC1 (dashed box). (B–G, I–L) Whole mount dorsal view of examples of the different constructs and effects of mutations. eGFP expression (green) indicates enhancer activity in electroporated (red) cells. (B,C) NC1.1 directs expression of eGFP in the same pattern as full-length NC1. (D,E) NC1.2 drives weak expression of eGFP in cranial neural crest. (F,G) NC1.1 M7 only drives weak eGFP expression in a small number of cells in no discernable pattern. (I, J) NC2.9 directs expression of eGFP in the same pattern as full-length NC2. (K,L) NC2.9 M11, containing a deletion of the Zic site, fails to drive eGFP in the neural crest. nc: neural crest, R: rhombomere.

The dynamic expression driven by the enhancer NC1 and its early activation, correlating with the onset of endogenous FoxD3, led us to explore upstream regulators and their binding motifs within NC1, responsible for early activation in the premigratory neural crest. To this end, conservation across vertebrates was used as a guide to identify putative core regions within the enhancer. The central region of NC1 was highly conserved with human, mouse and *Xenopus*, but showed no sequence conservation with zebrafish. Primers were designed to amplify fragments of NC1, which were tested for activity at stages HH9–10, corresponding to the time it drove strongest expression. Using this approach, NC1 was reduced to 553 bp (NC1.1) without loss of activity (Figure 4A, 4B). A further deletion to 303 bp (NC1.2) resulted in weak eGFP expression specifically in the cranial neural crest (Figure 4A, 4D), suggesting that the regions at the ends of NC1.1 amplify activity of the enhancer, although the critical regions are present within NC1.2. The sequence of NC1.2 was further analyzed by substituting 100 bp regions of sequence with eGFP coding sequence within the NC1.1 fragment. eGFP coding sequence was chosen as a random sequence to substitute for enhancer regions, so that the size and spacing was maintained, but did not alter expression in control experiments. This analysis revealed that 200 bp was required for expression mediated by the enhancer (Figure 4A). We then substituted 20 bp blocks of sequence with eGFP coding sequence across this region within NC1.1. This analysis revealed a region of 80 bp that was critical for detectable expression of eGFP (Figure 4A). An adjacent 92 bp region was required as a unit for eGFP expression; however substitutions of 20 bp blocks within this secondary region weakened but did not eliminate eGFP expression. None of the substitutions resulted in expansion of enhancer-driven expression. The 172 bp fragment (NC1.3) containing the most critical and supportive regions was amplified and electroporated into embryos, and the 80 bp putative core region (NC1.4) was tested by placing two copies in tandem into the ptkeGFP construct. NC1.3 alone drove very weak expression of eGFP in the neural crest. Interestingly, the NC1.4 cancatamer was sufficient to drive eGFP expression in the same pattern as the full-length NC1 enhancer, albeit slightly weaker, suggesting that the 80 bp NC1.4 fragment contains the core elements essential for activity of this enhancer.

Potential transcription factor binding sites within the core region were identified using Rvista and Jaspar databases (Figure S2A). Mutations were made to these sites by substituting 6–8 bp of the core binding site (marked in red or blue in Figure S2A). Mutations to the Ikaros binding site or to the Ets/Zeb binding site did not affect expression of eGFP (Figure S2B and S2C). In contrast mutation of the homeodomain site (Figure S2D and S2E) or Elk/Ets site resulted in loss of eGFP expression. Additionally, mutation of an Msx site (Figure S2A) reduced activity of the enhancer. Pax7, Msx1 and Msx2 are neural plate border genes expressed in the neural folds prior to expression of FoxD3, and candidates for direct activators of *FoxD3*. All of these can potentially bind to the homeodomain sites. Furthermore, Ets1 is expressed specifically in the cranial neural crest concomitant with the onset of FoxD3 expression.

### Dissection of NC2 reveals Zic binding sites are critical for enhancer activity

Similar to the dissection of NC1, we performed a series of deletions and substitutions to identify the core structure critical for activity of the NC2 enhancer (Figure 4H). NC2 is highly conserved in mouse, human, *Xenopus* and zebrafish. Stepwise deletions revealed a fragment, termed NC2.9, with similar albeit weakened activity to that seen with NC2 in the vagal and trunk neural crest, as well as weak activity in cranial migratory neural crest (Figure 4H, 4I). Subsequently, 100 bp and 30 bp substitutions were made within NC2.9, narrowing the essential regions of the enhancer to approximately 120 bp, encompassing a 90 bp core region surrounded by auxiliary regions required for strong expression (Figure 4H). Several deletions of the NC2 enhancer resulted in eGFP expression in the developing retina (NC2.6, NC2.9 M20) and otic vesicle (NC2.7), and using the full-length NC2 enhancer to drive eGFP, occasional weak expression could also be seen in these structures.

Importantly, deletion of the Zic site within the 90 bp critical core region resulted in complete loss of activity of the enhancer (Table S1). The auxillary (amplifying) region contains Pax, Ets and SoxE sites. Deletion of Pax or SoxE binding sites in the auxiliary region caused loss of NC2 activity in cranial neural crest, but did not affect vagal/trunk NC2 activity (Table S1). Similarly, deletion of an Ets1 site in the auxiliary region (M20), abolished activity in R1–R3 of the cranial neural crest, but did not affect vagal/trunk activity (Table S1). The results suggest that the NC2 enhancer itself is differentially regulated in the cranial neural crest versus trunk neural crest.

### Knock-down of potential regulators reveals differential control of NC1 and NC2

We next tested whether the putative transcription factors implicated by enhancer dissections could regulate enhancer driven reporter expression. To this end, individual embryos were electroporated on one side with FITC-conjugated control morpholino plus enhancer directing Cherry expression and with FITC-conjugated blocking morpholino plus enhancer-Cherry on the contralateral side.

**Figure 5:**
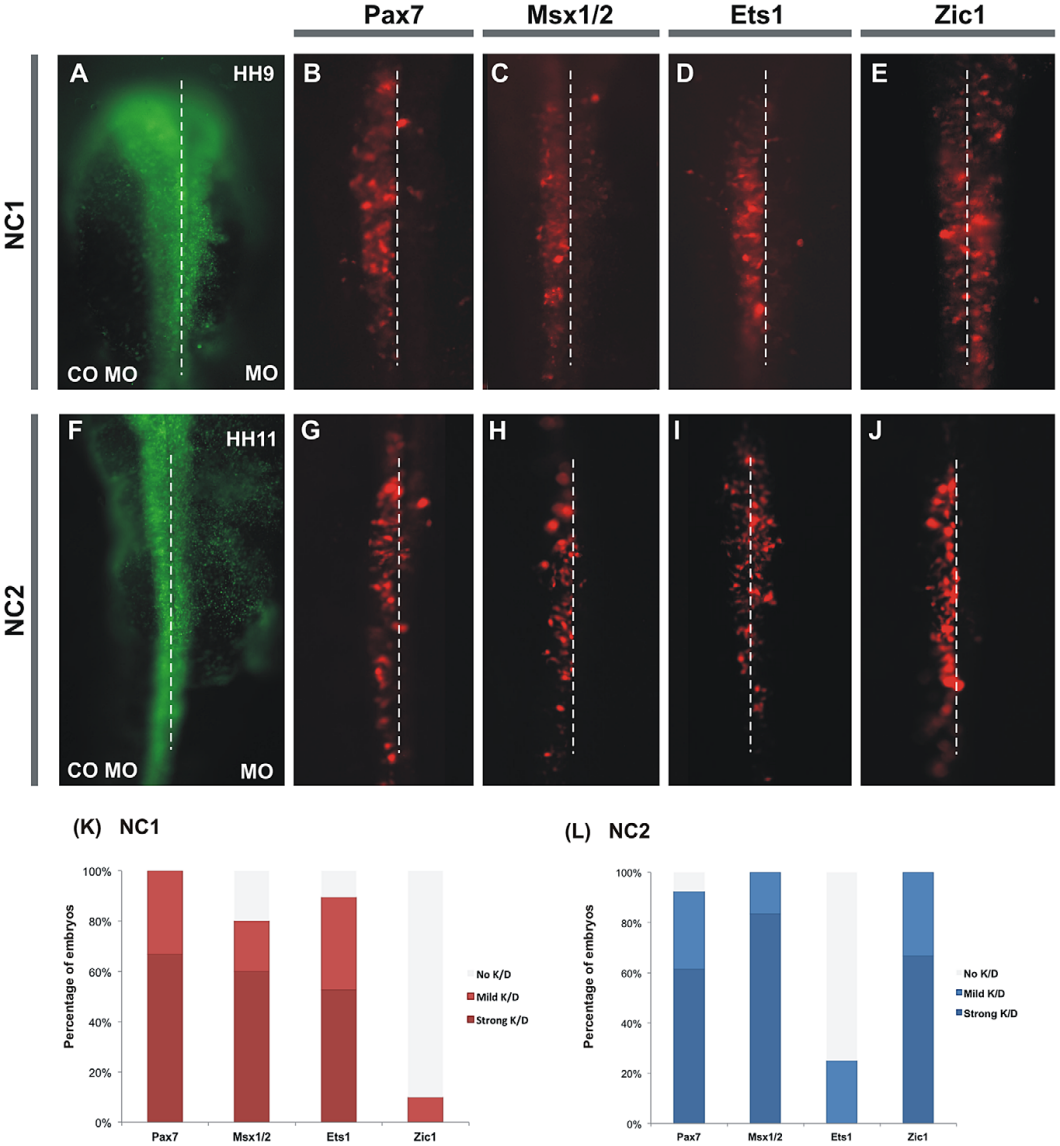
Knockdown of several putative inputs affects activity of NC1 and NC2 enhancers. (A,F) The left side of each embryo was electroporated with control morpholino plus a construct containing either NC1 (A–E) or NC2 (F–J) driving Cherry. The right side of each embryo was electroporated with the same construct plus indicated antisense morpholino. Knockdown of Pax7 (B), Msx1+2 (C), or Ets1 (D) results in dramatic loss of NC1.1 activity, whereas Zic1 morpholino (E) has no effect. In contrast, Pax7 (G), Msx1/2 (H) and Zic1 (J) have a strong effect on activity of the NC2 enhancer in the trunk, while Ets1 has no effect (I). (K, L) Percentage of embryos that showed either mild or strong reduction of Cherry expression on the side electroporated with antisense morpholino.

For NC1, morpholino-mediated loss of Pax7 (Figure 5B, 5K) protein resulted in significant loss of reporter expression on the target morpholino side (right). Whereas Msx1 knock-down alone resulted in a mild loss of Cherry expression and Msx2 knock-down had almost no phenotype (data not shown), the double MO knock-down exhibited a strong loss of reporter expression (Figure 5C, 5K). Additionally, knock-down of the transcription factor Ets1 resulted in strong loss of NC1 enhancer activity (Figure 5D, 5K). In contrast, morpholinos against other neural crest or neural plate specifiers like Zic1 (Figure 1E), Sox9 or AP-2 failed to alter NC1 reporter expression. These findings support the possibility that Pax7, Msx1/2, and Ets1 are direct inputs into the NC1 enhancer.

To confirm that the loss of Cherry positive cells was not due to loss of neural crest cells on the morpholino-treated side of the embryo, we examined other neural crest markers in embryos in which enhancer-driven Cherry expression was depleted (Figure S3). At the concentration of morpholinos used, we observed little change in *Sox9* expression (Figure S3G–S3I), demonstrating that the neural crest population was present in morpholino-treated embryos. Similarly, immunostaining with the HNK-1 antibody at stage HH10 confirmed the persistence of neural crest cells after morpholino treatment (Figure S3J–S3L).

We next examined the effects of knocking down putative regulators on expression driven by the NC2 in the vagal/trunk neural crest. Electroporation of both Pax7 and Msx1/2 morpholinos resulted in loss of NC2 activity in the trunk neural crest (Figure 5G, 5H, 5L) similar to the effects observed for NC1 activity in the cranial crest. In addition, electroporation of the Zic1 morpholino caused strong loss of NC2 activity specifically in the trunk (Figure 5J, 5L), suggesting this transcription factor is a key player in the regulation of trunk expression of FoxD3. On the other hand, Ets1 knock-down had no affect on trunk activity of NC2, which is not surprising given that this transcription factor is not expressed in the posterior neural crest (Figure 5I, 5L). Taken together, these results place Pax7 and Msx1/2 as general regulators of FoxD3 expression, while Ets1 and Zic1 seem to specifically regulate NC1 and NC2, respectively.

### Endogenous FoxD3 activity is affected by knockdown of Pax7, Ets1, Msx1/2, and Zic1

**Figure 6:**
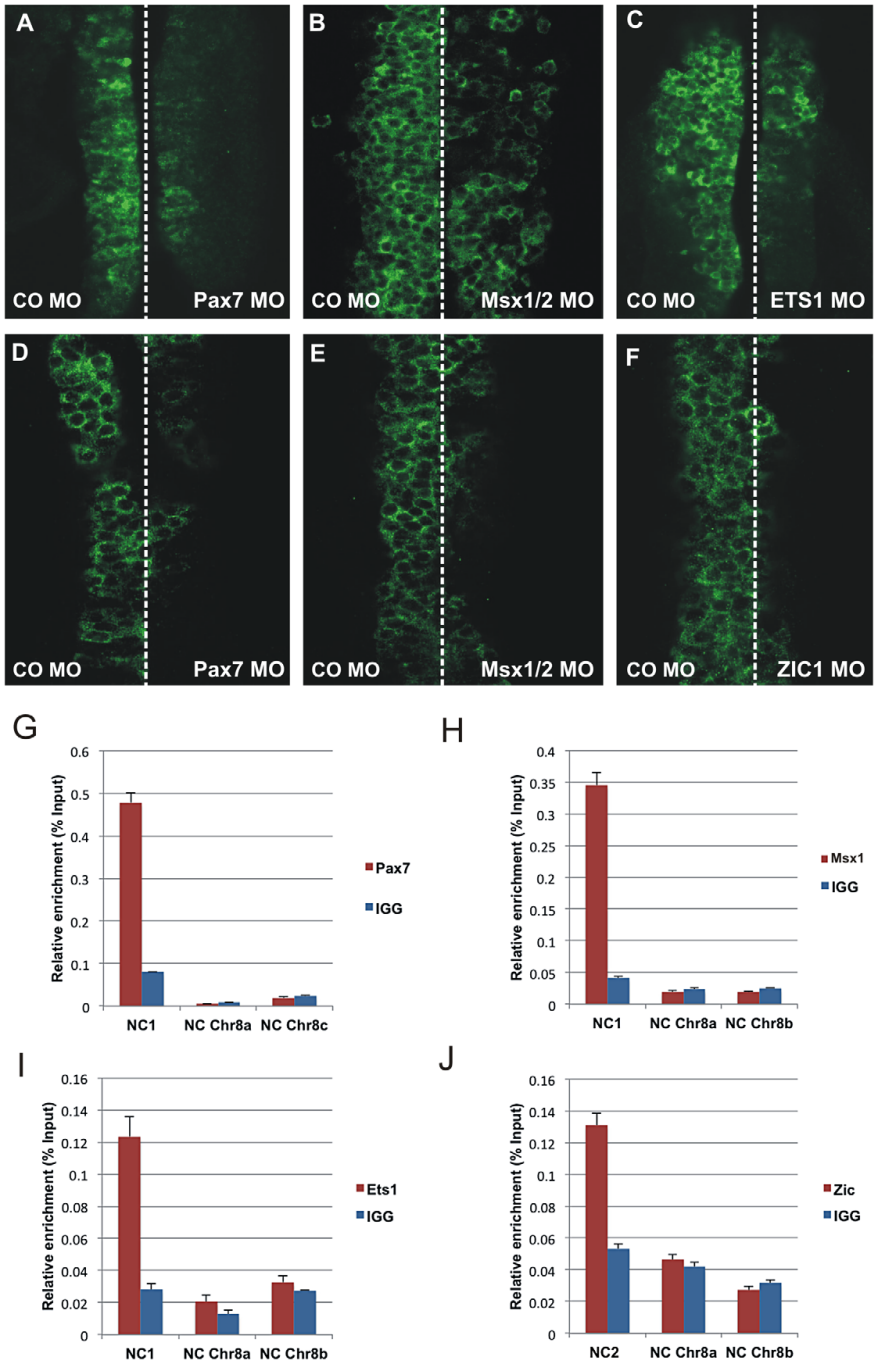
Effect of morpholino-mediated knockdown and chromatin immunoprecipitation. (A–I) Effect of morpholino-mediated knockdown of Pax7, Msx1/2, Ets1 and Zic1 on the endogenous expression of FoxD3. Knockdown of Pax7 (A), Msx1/2 (B) and Ets1 (C) results in reduction of endogenous expression of FoxD3 in the cranial neural crest. In trunk neural crest cells, electroporation of morpholinos to Pax7 (D), Msx1/2 (E) and Zic1 (F) results in reduction of FoxD3 expression. (G–J) Chromatin immunoprecipitation shows direct binding of Pax7, Ets1, Msx1 to the NC1 enhancer and Zic1 to NC2 enhancer. Immunoprecipitation of chromatin isolated from the midbrain dorsal neural tubes of chicken embryos using Pax7 (G), Msx1 (H) or Ets1 (I) antibodies was used in site-specific QPCR, with primers designed to amplify fragments within the NC1 region. The results reveal significant enrichment of the NC1 region amplicon, expressed as a percent of the total input chromatin, compared to negative control regions. (J) Immunoprecipitation of chromatin isolated from the trunk dorsal neural tubes of chicken embryos using Zic1 antibody reveals direct binding of this transcription factor to the NC2 enhancer compared to negative control regions.

To examine the effects of these regulators on endogenous gene expression, we performed morpholino-mediated loss-of-function of Pax7, Ets1, Msx1/2 and Zic1 and examined endogenous FoxD3 expression in newly forming cranial and trunk neural crest cells. Detection of FoxD3 was assessed by hybridization chain reaction (HCR), which reflects transcript levels more accurately than *in situ* hybridization and at subcellular resolution [19]. We found that morpholino mediated knock-down of Msx1/2, Pax7 and Ets1 caused a significant loss of cranial FoxD3 expression (Figure 6A–6C) at stage HH9, but not Sox9 or HNK-1 expression.

At trunk levels, knock-down of Msx1/2, Pax7 and Zic1 resulted in a significant reduction of endogenous FoxD3 expression (Figure 6D–6F), whereas loss of Ets1 had no effect. The results show that Pax, Msx and Zic transcription factors are not only important for mediating enhancer activity but also for endogenous expression of FoxD3 in the vagal/trunk neural crest.

### Pax7, Ets1, Msx1/2, and Zic1 directly regulate FoxD3 through the NC1 and NC2 enhancers

To demonstrate *in vivo* association of Pax7, Msx1 and Ets1 transcription factors with the NC1 enhancer, we performed quantitative chromatin immunoprecipitation (ChIP) experiments. Cross-linked chromatin isolated from the midbrain dorsal neural tube of HH8–9 embryos was immunoprecipitated using Pax7, Msx1 and Ets1 antibodies and ChIP-enriched DNA was used in site-specific qPCR, with primers designed to amplify fragments within the NC1 region. For all three factors, we found significant enrichment of the NC1 region amplicon, expressed as a percent of the total input chromatin, compared to IgG controls (Figure 6G–6I). No enrichment was detected in the negative control regions in chromosome 8 (Figure 6G–6I), confirming they are direct inputs into NC1. These data demonstrate that Pax7, Msx1 and Ets1 bind *in vivo* to the NC1 enhancer element in the cranial neural crest.

Given the striking effects of Zic1 knockdown on NC2 activity in the trunk region, we hypothesized that this transcription factor directly binds this enhancer in vivo. To examine this, we dissected dorsal trunk neural tubes of stage HH12 embryos, crosslinked and immunoprecipitated chromatin with a Zic1 antibody. The results show significant enrichment of the NC2 region amplicon, expressed as a percent of the total input chromatin, compared to IgG (Figure 6J). The results confirm that Zic1 directly associates with the NC2 enhancer in trunk neural crest.

Taken together, these results reveal direct transcriptional regulators of FoxD3 in the neural crest GRN, and highlight the differential regulation of FoxD3 in the cranial and trunk neural crest cells.

## Discussion

As proposed in a putative gene regulatory network [3], FoxD3 is predicted to be downstream of neural plate border specifier genes such as Msx1/2, Pax3/7 and Zic1. Indirect support for this regulatory connection comes from several previous studies. Mice null for *Pax3* lack expression of FoxD3 in the neural crest [13]. Knockdown of several genes in *Xenopus*, including Msx1, Pax3 and Zic1 results in loss of FoxD3 expression in the neural crest [20]–[22]. Similarly, knockdown of these genes and others expressed at the neural plate border in lamprey result in loss of FoxD3 expression [23]. Conversely, misexpression of these genes can induce expression of FoxD3 and other neural crest markers in *Xenopus* [20]–[22]. However, little was known about direct binding of any of these potential upstream transcription factors to a regulatory region of FoxD3, or the exact placement of these genes in relation to FoxD3 within the neural crest gene regulatory network. Importantly, these studies did not consider that FoxD3 may be differentially regulated at different axial levels.

### Multiple enhancers regulate dynamic FoxD3 expression in the neural crest

Our results suggest that expression of FoxD3 is regulated in the avian neural crest by at least two enhancers, which direct expression in largely distinct spatiotemporal domains (head versus vagal/trunk regions), as well as in different subpopulations of the cranial neural crest. The enhancer NC1 is active in premigratory and some migratory cranial neural crest rostral to R3, while enhancer NC2 activity initiates in a single continuous wave caudal to rhombomere 4, including vagal and trunk regions, but also later in a subpopulation of migrating cranial neural crest. In our analysis of the conserved regions within the FoxD3 locus, only these two regions were able to mediate reporter expression in patterns reflecting the distribution of neural crest. The proximity of the NC1 and NC2 enhancers to the FoxD3 coding region, the recapitulation of endogenous FoxD3 expression by the combined activity of the enhancers, and the effect of manipulating upstream regulators on both enhancers and endogenous FoxD3 expression, strongly suggest that NC1 and NC2 act as enhancers regulating endogenous expression of FoxD3 in the neural crest.

Comparison of the activity of these two enhancers with the cranial Sox10 enhancer Sox10E2 [5] using time-lapse imaging demonstrated for the first time that there is dynamic regulation of multiple enhancers within a population of cranial neural crest cells. We observed that the activity of the cranial NC1 enhancer is initially restricted to cells in the dorsal neural tube; only later is it activated *de novo* in actively migrating cranial neural crest cells, where its activity is preceded by that of Sox10E2. NC2 is active in only a few delaminating/emigrating cranial neural crest cells but in a majority of the migrating neural crest population. Interestingly, there is little overlap of NC1 and NC2 activity in the cranial neural crest, whereas both overlap with Sox10E2, which appears to be active in all of the migrating cranial crest population.

The minimal overlap in activity of NC1 versus NC2 in cranial neural crest populations raises the interesting possibility that there may be a regulatory switch of enhancers from NC1 to NC2 at the endogenous promoter of FoxD3 when the cells reside within the dorsal neural tube and/or are emigrating. Such competition at the promoter could occur if only a single enhancer can be functional at any given time on the FoxD3 promoter. If this is the case, the very few double labeled cells expressing NC1- and NC2-driven reporter expression may represent perdurance of eGFP protein rather than the actual levels of enhancer activity. The finding that NC1 and NC2 enhancers are active in generally separate cranial neural crest populations further suggests that the cranial neural crest represents a heterogeneous population, even as the cells are delaminating from the neural tube, and that this heterogeneity may be encrypted at the regulatory level.

It is intriguing to speculate that the differential activity of NC1 and NC2 in distinct subpopulations may reflect differential cell fate and commitment status of future neural crest derivatives. Consistent with the possibility that NC1 and NC2 activity may reflect commitment to different lineages, NC2 is later active in neural crest-derived dorsal root and trigeminal ganglia, whereas NC1 is active transiently in the branchial arches, but not in peripheral ganglia.

The activity of NC2 in the vagal and trunk neural crest recapitulated expression of endogenous FoxD3 in premigratory and migratory neural crest cells. In addition, FoxD3 is retained by a subset of neural crest derivatives [16]. Consistent with this, conditional knockout of FoxD3 in neural crest cells using the Wnt1-Cre line suggests that FoxD3 is required to maintain neural crest progenitors and that its loss biases their derivatives toward a mesenchymal fate at the expense of neural derivatives [24]. Thus, it appears to regulate the switch between neural/glia and melanocyte lineages [16].

NC2 not only was active in neuronal derivatives, but also directed activity in neural crest cells migrating along the dorsolateral pathway, which are melanocyte precursors that migrate 24 h after the ventrolateral population migrate to the ganglia. Cells on the dorsolateral population do not normally express FoxD3 [7]. Thus, NC2 likely is missing a repressor region for the pigment lineage that is present in the endogenous regulatory region. In fact, ectopic expression of FoxD3 in melanoblasts inhibits their migration onto the dorsolateral pathway, while down-regulation of FoxD3 results in premature dorsolateral migration and increases melanocyte differentiation in cultured neural crest [7]. FoxD3 represses transcription of Mitf, a key transcription factor required for melanocyte development [16], [25]. Our finding of an active enhancer in melanoblasts suggests that FoxD3 is normally repressed in melanoblasts, and this repression does not occur within the NC2 region. In the zebrafish *histone deacetylase 1* (*hdac1*) mutant, a severe loss of *mitfa* positive melanophores can be rescued by partial reduction of FoxD3; suggesting *hdac1* is required to repress FoxD3 in melanophores [25]. It is not yet clear whether this repression is direct or indirect and if it is conserved across species.

### At cranial levels, FoxD3 is regulated by Pax7 and Msx1/2 and Ets1, while trunk expression is dependent on Zic1

The current results establish for the first time a direct regulatory connection between the neural plate border genes, Pax7 and Msx1/2, and FoxD3, suggesting it is an immediate downstream target. This confirms and validates previous indirect evidence in *Xenopus*, lamprey and mouse, and provides further support for a conserved gene regulatory network in the neural crest. Pax7 and Pax3 are closely related paralogs which have overlapping expression and function [26]. Pax3 and Pax7 bind identical DNA binding domains, and while they show equal affinity for binding to the paired domain, Pax7 shows a higher affinity for the homeobox domain [27]. Both Pax3 and Pax7 are expressed in the developing neural crest, but in overlapping and distinct regions of the neural crest, and these patterns differ between species. In mouse and *Xenopus*, Pax3 is expressed in premigratory neural crest along the neural axis, and Pax7 is restricted to cranial levels (and very weak in *Xenopus*) [13], [28], [29]. In chick and zebrafish, Pax7 is expressed throughout the developing crest, and whereas Pax3 expression in neural crest is restricted to trunk levels in chick, in zebrafish it is also seen at cranial levels [4], [30], [31]. Evidence from *Xenopus,* mouse and lamprey suggests that Pax3 and/or Pax7 is required for FoxD3 expression and neural crest specification [13], [22]. In chick, Pax7 but not Pax3 knock-down at gastrula stages depletes neural crest specifier expression [4]. Mouse Pax3 mutants have a neural crest phenotype, and lack expression of FoxD3 in the trunk neural crest. However at cranial levels, where Pax7 is expressed, FoxD3 also is expressed [13]. Pax7 mutant mice have some craniofacial abnormalities, but survive well [28], and the impact of Pax3/Pax7 combined knockout on the neural crest has not been described. Substitution of Pax3 by Pax7 rescues the development of the neural crest [26], suggesting that there is partial redundancy between Pax3 and Pax7 in the mouse neural crest. In *Xenopus*, Pax3 is necessary for expression of FoxD3 [22], and in lamprey the Pax3/7 gene is similarly necessary for expression of the FoxD3 homolog FoxD-A [23].

Msx1 has been proposed to lie upstream of Pax3, FoxD3 and Snail2 during neural crest induction in *Xenopus* [21]. Loss of Msx1 or Msx2 in mice causes craniofacial abnormalities [32], [33], while the combined loss resulted in major defects in cranial neural crest derivatives, including mispatterning or reduction in size of cranial ganglia, loss, hypoplasticity or malformation of cranial bones, and conotruncal abnormalities [34]. Ablation of FoxD3 in mice in neural crest using Wnt-cre causes a similar phenotype at cranial levels; loss or reduction of many craniofacial structures, reduction in the size of cranial ganglia, subtle cardiac neural crest defects and also reduction in dorsal root ganglia size, and loss of enteric neural crest [24]. Cranial neural crest cells are still capable of undergoing migration in the absence of FoxD3 or Msx1/2, but many undergo apoptosis; in FoxD3 mutants apoptosis was seen in the neural tube or during migration [24], and in Msx1/2 mutants in the trigeminal ganglia and branchial arches [34]. As yet, the expression pattern of FoxD3 in the Msx1/2 mouse mutants is not known; however the strong similarities between the FoxD3 and Msx1/2 mutants at cranial levels provide support to the idea that Msx1/2 are immediately upstream of FoxD3 in the cranial neural crest. Differences at cranial levels between the mutant mice may reflect other roles of Msx genes, such as in neural tube and bone development. Other differences between the phenotypes suggest that in mice, Msx1/2 is not critical for neural crest development at trunk levels, unlike FoxD3. Although Msx transcription factors have been primarily described as transcriptional repressors [35], there is growing evidence for their role as transcriptional activators as well [36], [37]. Our results demonstrate that during avian cranial neural crest specification, Msx1/2 act as transcriptional co-activators of FoxD3.

Our data also show that Ets1 is necessary for initial FoxD3 expression since electroporation of Ets1 morpholinos during gastrulation (at HH5) depletes FoxD3 expression at HH9. In contrast, a dominant-negative Ets1 inhibited cranial neural crest migration but did not result in decreased FoxD3 expression [38]. Examination of the expression of FoxD3 and Ets1 by *in situ* hybridization suggests that Ets1 and FoxD3 are expressed concomitantly in the cranial neural crest. The difference in results between these two studies likely rests in the stages at which the knock-down reagents were effective, with the present results uncovering an earlier role for Ets1.

Recent work on the Sox10E2 enhancer showed that Sox10 expression in the cranial neural crest is regulated by Ets1, Sox9 and cMyb [5], [39]. The finding that Ets1 participates in activation of both FoxD3 and Sox10 at cranial level solidifies its potential crucial activation role in regulation of the cranial neural crest as a factor that initiates the specification module of the neural crest gene regulatory network. Interestingly, mis-expression of Ets1 in trunk levels confers cranial neural crest-like characteristics on trunk neural tube cells; namely increased delamination of neural crest independent of cell cycle phase [38]. This suggests that it plays a critical role in conferring head/trunk differences in the neural crest. However, conservation of this regulation across vertebrates remains to be determined. Ets1 is expressed by premigratory and migratory cranial neural crest in mice [40] and *Xenopus* [41]. Mice null for Ets1 have defects in cardiac neural crest, but none reported in cranial neural crest [40], [42]. Whether there is compensation for Ets1 in the cranial neural crest by other family members remains to be determined. Ets1 expression in chick neural crest is restricted to cranial levels; R4 and more rostral regions [38], [43]. Interestingly, the NC1 enhancer for FoxD3 is not active in R4, whereas the Sox10E2 enhancer is active to R6 [5], and Ets1 is active in R4 but not further caudally [38].

Although there is little published information regarding the molecular players are involved in the establishment of the more caudal neural crest populations, the present results implicate the neural plate border specifier, Zic1, as a critical factor in the control of FoxD3 expression at vagal and trunk levels. Zic1 has been shown to be required for FoxD3 expression in *Xenopus* neural crest [21], [22] where it is likely to partner with Pax3 in neural crest specification. Conversely, over-expression of Zic1 causes expansion of the FoxD3 and *Snail2* expression domains [22], albeit it is unclear whether this occurs via direct or secondary interactions. The role of Zic1 as a trunk specific activator of FoxD3 is corroborated by expression data (Simões-Costa M., unpublished observations) suggesting much higher Zic1 transcript levels in the vagal/trunk avian neural folds than at cranial levels at the onset of FoxD3 expression. Our results are consistent with complementary functions of Zic1 in trunk and Ets1 in cranial neural crest specification in the avian embryo.

The present study expands the number of known direct regulatory interactions in the cranial neural crest gene regulatory network, confirming a direct regulation of FoxD3 by Pax3/7 and Msx1/2, and revealing a previously unknown regulation of FoxD3 by Ets1. We have also identified Zic1 as a key player in setting up the FoxD3 expression domain in the trunk neural crest. Several other genes, like Hairy2, Sox10 and Sox5, have been suggested to regulate FoxD3 expression [44]–[46]; however it remains to be determined whether this regulation is direct or indirect.

### Differential control of head versus trunk neural crest

It is well known that the developmental potential of neural crest cells varies along different levels of the neural axis. Quail/chick chimeras have elegantly demonstrated that both the pathways of migration and derivatives differ depending upon the axial level from which neural crest cells emigrate [47]. For example, cranial but not trunk neural crest cells normally contribute to bone and cartilage. Similarly, vagal neural crest cells contribute to the enteric nervous system whereas other neural crest populations normally do not [48].

**Figure 7:**
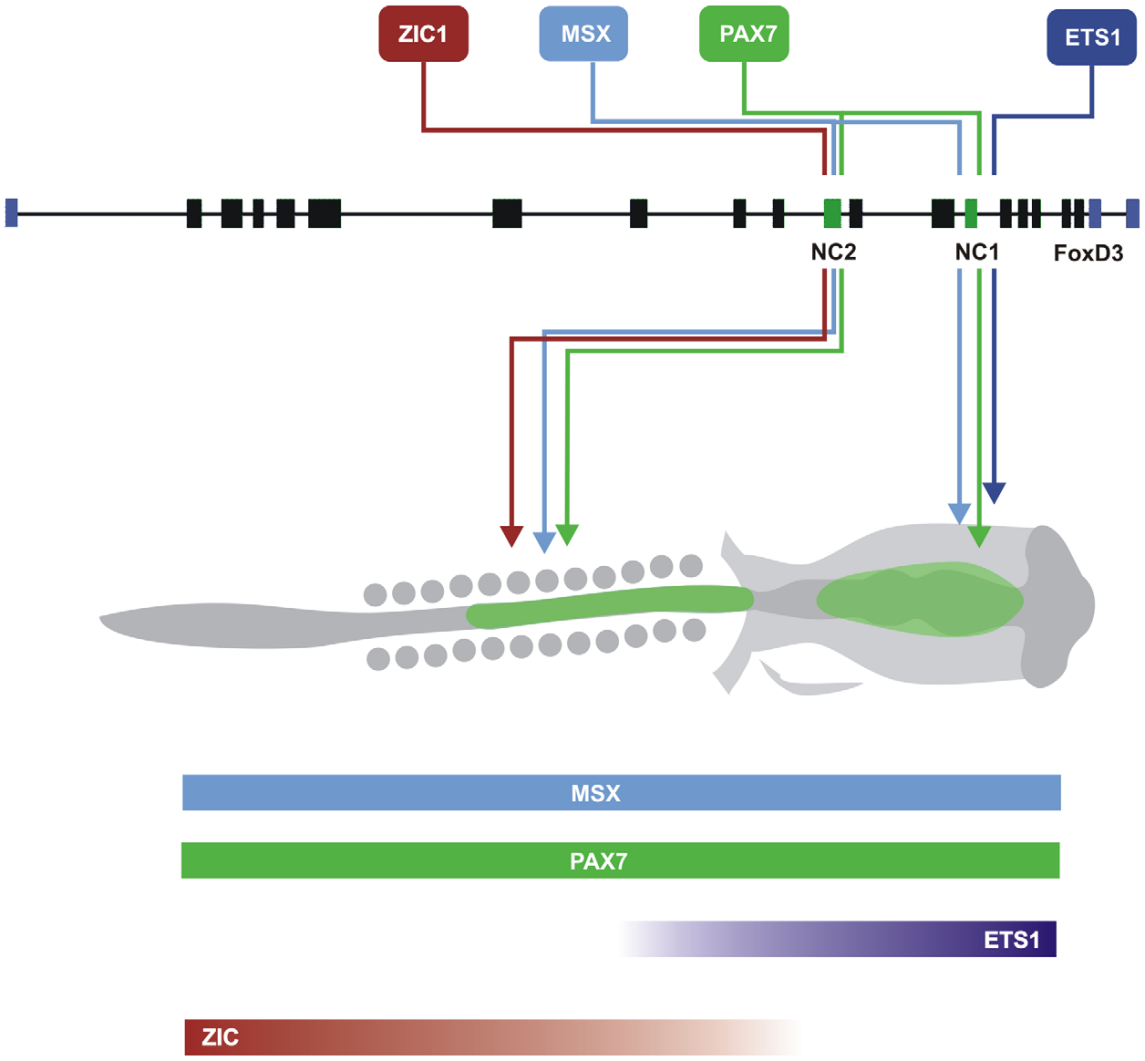
Model for differential regulation of FoxD3 in cranial and trunk neural crest cell populations. FoxD3 expression is controlled by distinct inputs and enhancers at different axial levels. Ets1 is critical for activating NC1 at cranial levels, while the neural plate border specifier, Zic1, is required for NC2 activity in the trunk. Both Zic1 and Ets1 transcription factors appear to act in concert with Pax7 and Msx1/2 that are expressed along the entire neural axis.

Our data show that the inputs to FoxD3 in the vagal/trunk region are distinct from those functioning at cranial levels, suggesting a model for region-specific expression of FoxD3 (Figure 7). Whereas the neural plate border specifier Zic1 appears to be a critical input for NC2 activity in the trunk, Ets1 is critical for activating NC1 at cranial levels. Both Zic1 and Ets1 transcription factors appear to act in concert with Pax7 and Msx1/2 which are expressed along the entire neural axis. To date, no transcription factors have been found to be selectively expressed in particular regions of the premigratory neural crest. However, the discovery of cranial-specific enhancers for FoxD3 (this study) and Sox10 [5] clearly suggest that these differences are inherent at the regulatory level. The existence of these enhancers supports the idea that both spatial and temporal information is encoded in the genome.

## Materials and Methods

### Enhancer constructs

The genomic region of chicken FoxD3 was compared to other vertebrates using the ECR Browser (http://ecrbrowser.dcode.org) and comparative analysis tracks of UCSC Genome Browser (http://genome.ucsc.edu/). We analyzed a 160 kb genomic region encompassing the FoxD3 locus up to the first upstream (Atg4C) and downstream (Alg6) of neighboring genes. Regions containing elements found to be highly conserved across most vertebrates including human, mouse and *Xenopus* were amplified using Expand High Fidelity Plus system (Roche, Indianapolis, IN) with CH261-166E22 and CH261-100C15 (CHORI BAC Resources, http://bacpac.chori.org) BAC clones as templates and directionally cloned into the ptkeGFP or ptkCherry vectors [5], [17]. Mouse neural crest enhancers mNC1 and mNC2 were amplified using Expand High Fidelity Plus system from genomic cDNA. For use in multiple enhancer time-lapse experiments, ptkCitrine and ptkCerulean plasmids were constructed by swapping the eGFP coding region by Citrine and Cerulean sequences, respectively. Appropriate enhancer elements (Sox10E from [5] and NC1, NC2 – this study) were cloned into ptkCherry, ptkCitrine and ptkCerulean, respectively.

### Electroporation and time-lapse imaging of slice cultures

Chicken embryos were electroporated at HH4 using previously described techniques [1], [49]. FITC-conjugated morpholinos (against target factors or control morpholino) at concentrations from 1–3 mM combined with 1 mg/ml of enhancer_reporter Cherry constructs were electroporated only on one half of the embryo. For morpholino knockdown of regulators, electroporations were performed at HH5 or HH5+ to avoid disruption of the neural plate border. Fifteen to twenty embryos were analyzed for each of the morpholinos used. To electroporate HH8–14 chicken embryos in ovo, previously described techniques [50] were used with minor modifications; both constructs were injected at a concentration of 2 mg/ml each, and embryos were electroporated with 5 30ms-square pulses of 22 V with 100 ms rest in between each pulse. After incubation, embryos were collected and fixed in 4% paraformaldehyde for 1 hour, then viewed using fluorescence microscopy. Images were captured using a Zeiss Axioskop 2 plus microscope with AxioVision 4.6 software, and compiled using Adobe Photoshop 7.0 and Adobe Illustrator 10. For dynamic multiple enhancer analysis in slice culture, embryos were electroporated at stage HH4 with three constructs simultaneously (Sox10E-Cherry/NC1-Citrine/NC2-Cerulean) to allow for proper spectral separation of reporter signals from different enhancers. After roughly 16 hours of incubation, cranial midbrain regions were prepared and imaged as described previously [18].

### *In situ* hybridization

Whole mount and section *in situ* hybridization for FoxD3 were performed using previously described procedures [51]. Whole mount *in situ* hybridization for eGFP was modified using the guidelines in [52]. Double fluorescence *in situ* hybridization was performed according to [53], and hybridization chain reaction (HCR) to detect endogenous FoxD3 was conducted according to [19].

### Antibody labeling and imaging

Some embryos expressing RFP and eGFP were processed and cryosectioned at 14 mm. Select sections were labeled using the HNK-1 antibody (diluted 1/50), secondarily detected using goat anti-mouse IgM Alexa 350 (1/200; Molecular Probes). For whole mount immunostaining we used the protocol described by [54] (FoxD3 antibody generously provided by Patricia Labosky and Michelle Southard-Smith).

### Enhancer substitutions and mutations

Regions of NC1 and NC2 were replaced with eGFP coding sequence using fusion PCR protocol. For 100 bp substitutions, the region of eGFP used was tggagtacaactacaacagccacaacgtctatatcatggccgacaagcagaagaacgg catcaaggtgaacttcaagatccgccacaacatcgaggacgg, for 30 bp substitutions acaagcagaagaacggcatcaaggtgaact and for 20 bp substitutions tggagtacaactacaacagc. Fragments were amplified using primers detailed in Table S2 and fused using the method adapted from [55]. Amplified fusion fragments were cloned into ptkeGFP and sequenced to ensure no additional mutations were present. ECR browser (http://rvista.dcode.org/) and Jaspar database (http://jaspar.genereg.net/cgi-bin/jaspar_db.pl) were used to predict and analyze binding motifs within highly conserved regions. Individual sites were mutated by substituting 6–8 adjacent critical base pairs with GFP coding sequence, using fusion PCR and sub-cloning into ptkeGFP. Primers used are listed in Table S3. Mutated enhancer constructs were electroporated into stage HH4 embryos as described above and analyzed for expression of eGFP and RFP at stages HH8–12. A minimum of five embryos was examined for each condition.

### Chromatin immunoprecipitation

ChIP was performed using chromatin prepared from dorsal neural tube regions of HH8–10 (4–10 somite) chicken embryos using Ets1 (sc-350;Santa Cruz), Pax7 (ab34360. Abcam) and Msx1 antibodies (Sigma M0944) with normal rabbit IgGs (sc-2027,Santa Cruz;ab27478, Abcam) as previously described [56]. For the Zic1 ChIP chromatin was isolated from the dorsal neural tube regions from the trunk of HH11 embryos. Immunoprecipitation was performed with a Zic1 antibody from Sigma (HPA004098).

## Supporting Information

Figure S1 Expression of NC2 in dorsal root ganglia, melanocytes and enteric nervous system. (A) *In situ* hybridization for FoxD3 showing expression in a dorsal root ganglion (DRG) adjacent to the neural tube (NT) in the trunk. No FoxD3 expression is present underneath the ectoderm where melanocytes are localized. (B) Transverse section through the trunk region similar to that shown in (A). eGFP (green) driven by enhancer NC2 is observed in the DRG as well as underneath the ectoderm (arrows) in presumptive melanocytes on the dorsolateral pathway. (C) Whole mount view of NC2 driven eGFP activity at HH19. Expression can be seen in the dorsal root ganglia (arrow) and melanoblasts (arrowheads). (D) *In situ* hybridization for eGFP shows expression in DRGs (arrow) and melanoblasts (arrowheads). (E) Transverse section of (D) confirms expression of eGFP in migrating melanoblasts (arrows). (F) NC2 activity can be seen in neural crest cells in the gut at HH27.
(TIF)

Figure S2 Putative transcription factor binding sites in the NC1 core region were subsequently mutated to examine effects on activity. (A) Core region of the enhancer with several binding sites highlighted. Mutation of sites in blue had no effect on the activity of the enhancer; sites in red abolished activity of enhancer when mutated. Results of two of the mutations are shown at HH9. eGFP expression (green) indicates activity of the enhancer in electroporated (red) cells. Faint background fluorescence can be seen in the neural tube and neural crest. (B,C) Mutation of the Ets/Zeb site did not abolish eGFP activity in the neural crest. (D,E) Mutation of the homeodomain (HD) site abolished activity in the cranial neural crest, resulting in a small number of cells weakly expressing eGFP.
(TIF)

Figure S3 Sox9 and HNK-1 expression in neural crest persists after knock-down of Pax7, Msx1/2 and Ets1 morpholinos. (A–C) Embryos in which NC1 enhancer-driven Cherry was depleted via knockdown of Pax7, Msx1/2 and Ets1 (see Figure 4) were analyzed for expression of neural crest markers, Sox9 and HNK-1 epitope, even though endogenous FoxD3 was down-regulated (D–F). Sox9 expression was only slightly reduced (G–I), indicating neural crest cells were present in morpholino-treated embryos. (J–L) Immunostaining with the HNK-1 antibody at stage HH10 confirmed the presence of neural crest cells after morpholino treatment.
(TIF)

Table S1 Mutational analysis of the NC2 enhancer reveals importance of Zic binding sites. Mutation M11, which impairs a Zic binding site, causes complete loss of trunk NC2 activity. Mutations M11, M15, M18 and M20 suppress cranial NC2 activity but only result in a slight reduction of enhancer expression in the trunk.
(DOCX)

Table S2 Primers used for NC1, NC2 deletions and substitutions. Text in capitals indicates enhancer sequence, and text in small letters indicates replacement GFP sequence. To make the mutated constructs, mutated primers were paired with flanking primers NC1.1 or NC2.9, amplified and joined in a fusion PCR reaction using the flanking primers NC1.1 or NC2.9.
(DOCX)

Table S3 Primers used for binding site mutations of NC1. Text in capitals indicates mutated sequence. To make the mutated constructs, primers were paired with flanking primers NC1.1, amplified and joined in a fusion PCR reaction using the flanking NC1.1 primers.
(DOCX)

Video S1 Dynamic regulation of FoxD3 and Sox10 enhancers in the cranial neural crest. Time-lapse movie shows differential temporal and spatial activity of NC1 (green), NC2 (blue) and the Sox10E2 (red) enhancers in a chick cranial slice preparation. NC1 drives expression of the reporter in the premigratory neural crest, preceding Sox10E enhancer activity. NC2 activity was observed in few cells within the neural tube, and few delaminating neural crest cells in which Sox10E2 is also active.
(M4V)

Video S2 Time lapse movie of migrating cranial neural crest cells electroporated with NC1:eGFP and NC2:Cherry. Time-lapse movie shows little overlap between cells with activity of NC1 and NC2. NC1 is active transiently in early neural crest cells, while NC2 seems to be primarily responsible for FoxD3 expression in migratory neural crest at cranial levels.
(M4V)
