# Supplementary figures and images for: Dynamic and Differential Regulation of Stem Cell Factor FoxD3 in the Neural Crest Is Encrypted in the Genome
Source: PLoS Genet. 2012 Dec 20;8(12):e1003142. doi: 10.1371/journal.pgen.1003142 (PMC3527204; doi:10.1371/journal.pgen.1003142)

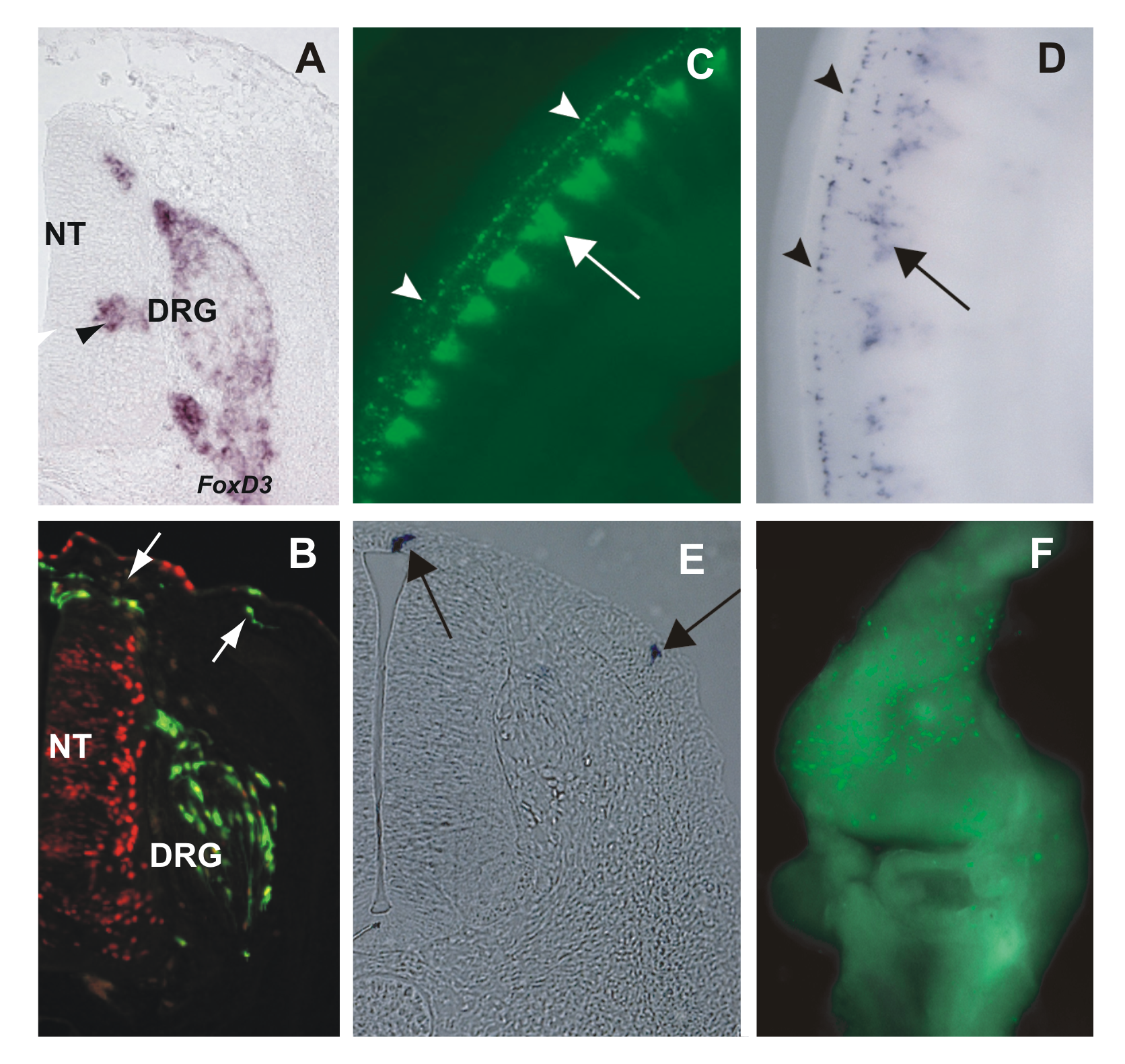

Supplement: Figure S1 — Expression of NC2 in dorsal root ganglia, melanocytes and enteric nervous system. (A) In situ hybridization for FoxD3 showing expression in a dorsal root ganglion (DRG) adjacent to the neural tube (NT) in the trunk. No FoxD3 expression is present underneath the ectoderm where melanocytes are localized. (B) Transverse section through the trunk region similar to that shown in (A). eGFP (green) driven by enhancer NC2 is observed in the DRG as well as underneath the ectoderm (arrows) in presumptive melanocytes on the dorsolateral pathway. (C) Whole mount view of NC2 driven eGFP activity at HH19. Expression can be seen in the dorsal root ganglia (arrow) and melanoblasts (arrowheads). (D) In situ hybridization for eGFP shows expression in DRGs (arrow) and melanoblasts (arrowheads). (E) Transverse section of (D) confirms expression of eGFP in migrating melanoblasts (arrows). (F) NC2 activity can be seen in neural crest cells in the gut at HH27. (TIF) [file pgen.1003142.s001.tif]

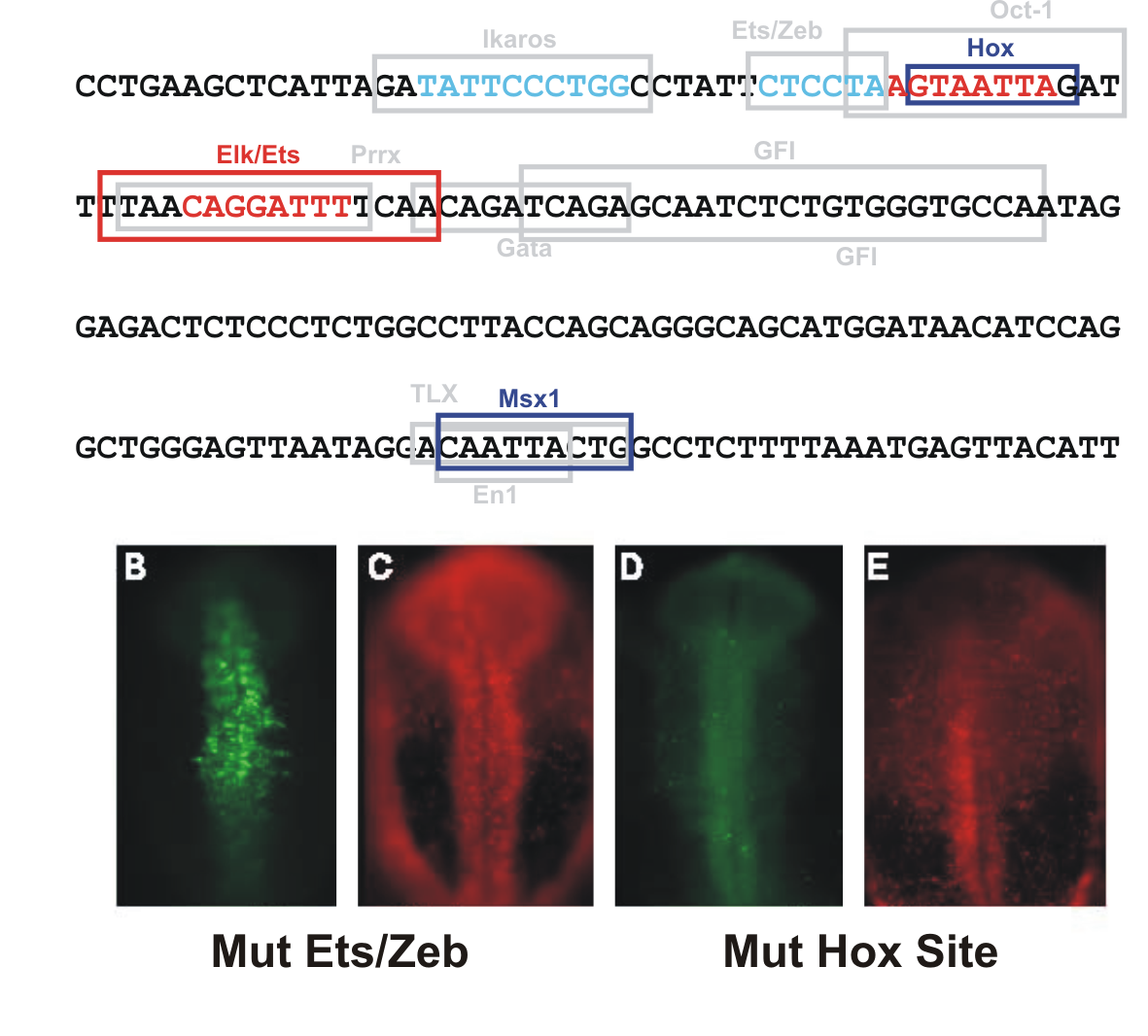

Supplement: Figure S2 — Putative transcription factor binding sites in the NC1 core region were subsequently mutated to examine effects on activity. (A) Core region of the enhancer with several binding sites highlighted. Mutation of sites in blue had no effect on the activity of the enhancer; sites in red abolished activity of enhancer when mutated. Results of two of the mutations are shown at HH9. eGFP expression (green) indicates activity of the enhancer in electroporated (red) cells. Faint background fluorescence can be seen in the neural tube and neural crest. (B,C) Mutation of the Ets/Zeb site did not abolish eGFP activity in the neural crest. (D,E) Mutation of the homeodomain (HD) site abolished activity in the cranial neural crest, resulting in a small number of cells weakly expressing eGFP. (TIF) [file pgen.1003142.s002.tif]

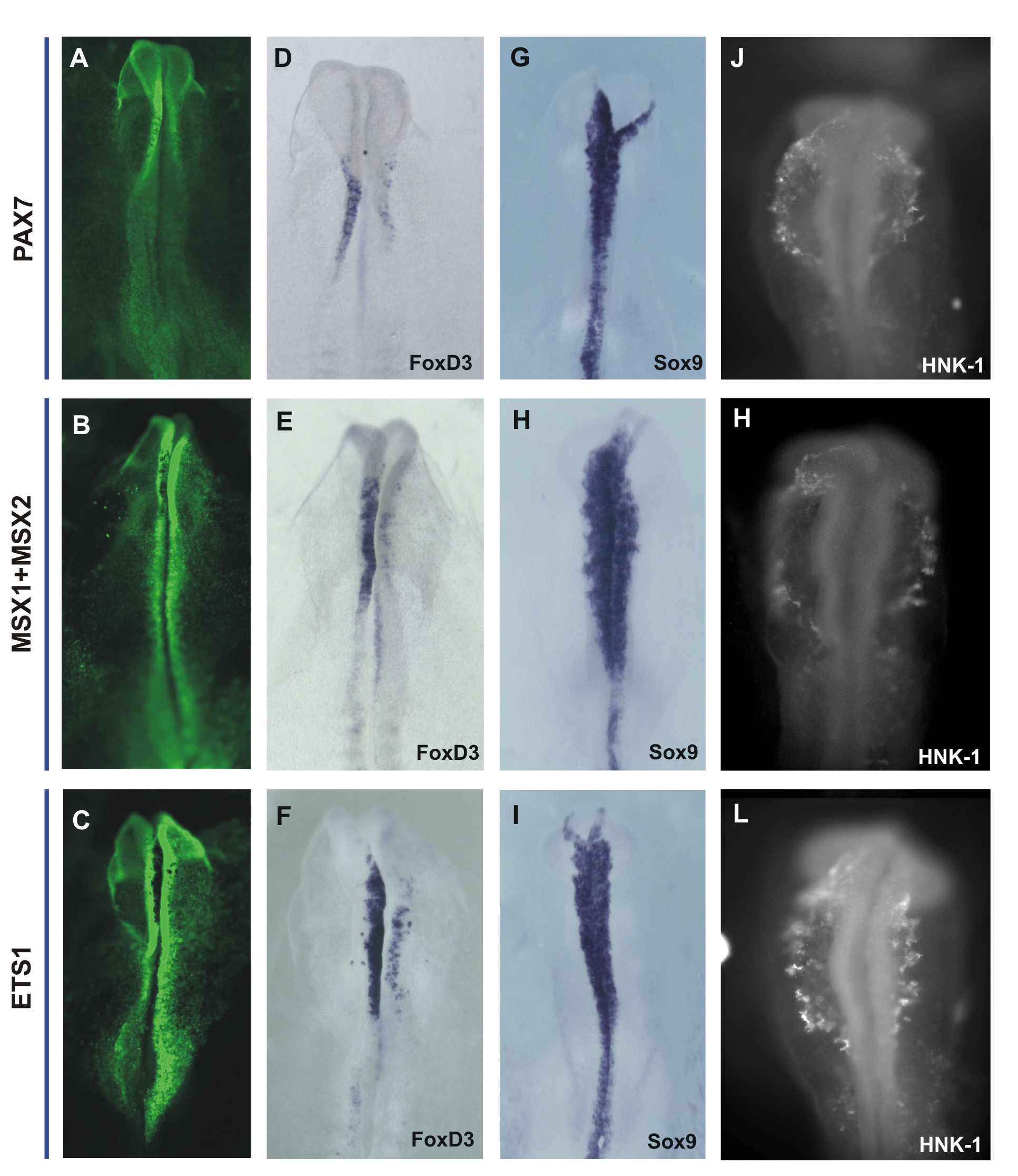

Supplement: Figure S3 — Sox9 and HNK-1 expression in neural crest persists after knock-down of Pax7, Msx1/2 and Ets1 morpholinos. (A–C) Embryos in which NC1 enhancer-driven Cherry was depleted via knockdown of Pax7, Msx1/2 and Ets1 (see Figure 4) were analyzed for expression of neural crest markers, Sox9 and HNK-1 epitope, even though endogenous FoxD3 was down-regulated (D–F). Sox9 expression was only slightly reduced (G–I), indicating neural crest cells were present in morpholino-treated embryos. (J–L) Immunostaining with the HNK-1 antibody at stage HH10 confirmed the presence of neural crest cells after morpholino treatment. (TIF) [file pgen.1003142.s003.tif]
