# Supplementary material for: Dynamic and Differential Regulation of Stem Cell Factor FoxD3 in the Neural Crest Is Encrypted in the Genome
Source: PLoS Genet. 2012 Dec 20;8(12):e1003142. doi: 10.1371/journal.pgen.1003142 (PMC3527204; doi:10.1371/journal.pgen.1003142)
Supplement: Table S1 — Mutational analysis of the NC2 enhancer reveals importance of Zic binding sites. Mutation M11, which impairs a Zic binding site, causes complete loss of trunk NC2 activity. Mutations M11, M15, M18 and M20 suppress cranial NC2 activity but only result in a slight reduction of enhancer expression in the trunk. (DOCX) [file pgen.1003142.s004.docx]

**Supplemental Table 1:** **Mutational analysis of the NC2 enhancer reveals importance of Zic binding sites.**

| Enhancer fragment | Location | Cranial NC | Vagal/Trunk NC | Binding sites |
| --- | --- | --- | --- | --- |
| NC2.9 | 761-1840 | ++ | +++ |  |
| NC2.9 M11 | ∆1124-1153 | - | - | Zic |
| NC2.9 M15 | ∆1244-1273 | - (+ R4) | ++ | Pax4/6  Pax8 |
| NC2.9 M18 | ∆1334-1363 | - | ++ | SoxE |
| NC2.9 M20 | ∆1394-1423 | - (+ R4) | ++ | Ets1 |
