# Supplementary material for: Dynamic and Differential Regulation of Stem Cell Factor FoxD3 in the Neural Crest Is Encrypted in the Genome
Source: PLoS Genet. 2012 Dec 20;8(12):e1003142. doi: 10.1371/journal.pgen.1003142 (PMC3527204; doi:10.1371/journal.pgen.1003142)
Supplement: Table S2 — Primers used for NC1, NC2 deletions and substitutions. Text in capitals indicates enhancer sequence, and text in small letters indicates replacement GFP sequence. To make the mutated constructs, mutated primers were paired with flanking primers NC1.1 or NC2.9, amplified and joined in a fusion PCR reaction using the flanking primers NC1.1 or NC2.9. (DOCX) [file pgen.1003142.s005.docx]

**Supplemental Table 2. Primers used for NC1, NC2 deletions and substitutions.**

| **Primer Name** | **Primer Sequence** |
| --- | --- |
| NC1 fwd | AGGCAATGCAGCGAATGACC |
| NC1 rev | gacctggctccctttgagac |
| NC1.1 fwd | CAGTAAGCTTTCCACCAACA |
| NC1.1 rev | gtttaacatacactatccaatg |
| NC1.2 fwd | CCTGAAGCTCATTAGATATT |
| NC1.2 rev | ccgacatttgggaaattaaa |
| NC1.3 fwd | CATTAGATATTCCCTGG |
| NC1.3 rev | AAAGAGGCCAGTAATTGTC |
| NC1 80bp core fwd | cattagatattccct |
| NC1 80bp core rev | cccacagagattgct |
| NC1.1 M1 fwd | caagcagaagaacggcatcaaggtgaacttcaagatccgccacaacatcgaggacgggagactctccct |
| NC1.1 M1 rev | gatgccgttcttctgcttgtcggccatgatatagacgttgtggctgttgtagttgtactccaaaacccactgaa |
| NC1.1 M2 fwd | caagcagaagaacggcatcaaggtgaacttcaagatccgccacaacatcgaggacggACTTAAGCTAAATGG |
| NC1.1 M2 rev | gatgccgttcttctgcttgtcggccatgatatagacgttgtggctgttgtagttgtactccactcctattggca |
| NC1.1 M3 fwd | caagcagaagaacgg catcaaggtgaacttcaagatccgccacaacatcgaggacggCGGATTTTGGTG |
| NC1.1 M3 rev | gatgccgttcttctgcttgtcggccatgatatagacgttgtggctgttgtagttgtactccacgattaatgtaactc |
| NC1.1 M4 fwd | agtacaactacaacagcCATTAGATATTCC |
| NC1.1 M4 rev | tgttgtagttgtactccaaagaattctctttt |
| NC1.1 M5 fwd | agtacaactacaacagcATTCTCCTAAGTA |
| NC1.1 M5 rev | tgttgtagttgtactccaagcttcaggaaa |
| NC1.1 M6 fwd | agtacaactacaacagcTAACAGGATTTTC |
| NC1.1 M6 rev | tgttgtagttgtactccaataggccaggga |
| NC1.1 M7 fwd | agtacaactacaacagcGAGCAATCTCTG |
| NC1.1 M7 rev | tgttgtagttgtactccataaaatctaattacttag |
| NC1.1 M8 fwd | agtacaactacaacagcAATAGGAGACTC |
| NC1.1 M8 rev | tgttgtagttgtactccatgatctgttgaaa |
| NC1.1 M9 fwd | agtacaactacaacagcTCTGGCCTTACC |
| NC1.1 M9 rev | tgttgtagttgtactccacccacagagatt |
| NC1.1 M10 fwd | agtacaactacaacagcAGCATGGATAAC |
| NC1.1 M10 rev | tgttgtagttgtactccagggagagtctccta |
| NC1.1 M11 fwd | agtacaactacaacagcTGGGAGTTAATAG |
| NC1.1 M11 rev | tgttgtagttgtactccagccctgctggtaa |
| NC1.1 M12 fwd | agtacaactacaacagcACTGGCCTCTTT |
| NC1.1 M12 rev | tgttgtagttgtactccagcctggatgtta |
| NC1.1 M13 fwd | agtacaactacaacagcTTACATTAATGCACT |
| NC1.1 M13 rev | tgttgtagttgtactccaaattgtcctattaac |
| NC1.1 M14 fwd | agtacaactacaacagcACTTAAGCTAAATG |
| NC2 fwd | TGAGTGTGCCTCCATGTGTC |
| NC2 rev | gatggtgcagcacacggttg |
| NC2.1/NC2.4 rev | tgtggtaggcttattgttttgct |
| NC2.2 rev | tcggttttgtttcacagtttg |
| NC2.3/NC2.4/NC2.6/NC2.9 fwd | GGTGCATAGAACAAACTGTG |
| NC2.5 fwd | GCACTGGGTTCATGAAGTTTC |
| NC2.5 rev | ctacctcagaaggcattgta |
| NC2.7/NC2.8 fwd | CGATTCTCTGTCTGCCAATTT |
| NC2.8/NC2.9/NC2.10 rev | gttcacccagtaaaccagta |
| NC2.10 fwd | TGTCATCTTCCGCTCACTT |
| NC2.9 M1 fwd | caagcagaagaacggcatcaaggtgaacttcaagatccgccacaacatcgaggacggGGAAACTGATGG |
| NC2.9 M1 rev | gatgccgttcttctgcttgtcggccatgatatagacgttgtggctgttgtagttgtactccagacataactttgtc |
| NC2.9 M2 fwd | caagcagaagaacggcatcaaggtgaacttcaagatccgccacaacatcgaggacggAAATTACTCCGATT |
| NC2.9 M2 rev | gatgccgttcttctgcttgtcggccatgatatagacgttgtggctgttgtagttgtactccaccaaatactttcact |
| NC2.9 M3 fwd | caagcagaagaacggcatcaaggtgaacttcaagatccgccacaacatcgaggacggTAGCAAGGGGCTT |
| NC2.9 M3 rev | gatgccgttcttctgcttgtcggccatgatatagacgttgtggctgttgtagttgtactccagtatcatttcaattag |
| NC2.9 M4 fwd | caagcagaagaacggcatcaaggtgaacttcaagatccgccacaacatcgaggacggCCGCTACCTTCA |
| NC2.9 M4 rev | gatgccgttcttctgcttgtcggccatgatatagacgttgtggctgttgtagttgtactccacagcagccactt |
| NC2.9 M5 fwd | caagcagaagaacggcatcaaggtgaacttcaagatccgccacaacatcgaggacggGTATTCATCCCCAA |
| NC2.9 M5 rev | gatgccgttcttctgcttgtcggccatgatatagacgttgtggctgttgtagttgtactccatggtaggcttat |
| NC2.9 M6 fwd | caagcagaagaacggcatcaaggtgaacttcaagatccgccacaacatcgaggacggCAGTAGGAAAAAC |
| NC2.9 M6 rev | gatgccgttcttctgcttgtcggccatgatatagacgttgtggctgttgtagttgtactccaaacacttatctctac |
| NC2.9 M7 fwd | caagcagaagaacggcatcaaggtgaacttcaagatccgccacaacatcgaggacggTAGTTCAACTGTGT |
| NC2.9 M7 rev | gatgccgttcttctgcttgtcggccatgatatagacgttgtggctgttgtagttgtactccaaaccgagcgcaa |
| NC2.9 M8 fwd | caagcagaagaacggcatcaaggtgaacttcaagatccgccacaacatcgaggacggTCAGTGCAATTC |
| NC2.9 M8 rev | gatgccgttcttctgcttgtcggccatgatatagacgttgtggctgttgtagttgtactccagaactattttggat |
| NC2.9 M9 fwd | gaagaacggcatcaaggtgaactatgggcaataat |
| NC2.9 M9 rev | ttgatgccgttcttctgcttgtccaaatactttca |
| NC2.9 M10 fwd | gaagaacggcatcaaggtgaactacctccctgtta |
| NC2.9 M10 rev | ttgatgccgttcttctgcttgtggcaaccaaact |
| NC2.9 M11 fwd | gaagaacggcatcaaggtgaactgaaatgatacaaat |
| NC2.9 M11 rev | ttgatgccgttcttctgcttgtcatgacttttttg |
| NC2.9 M12 fwd | gaagaacggcatcaaggtgaactctgccaatttag |
| NC2.9 M12 rev | ttgatgccgttcttctgcttgtaattagttactagc |
| NC2.9 M13 fwd | gaagaacggcatcaaggtgaactggtcaaatgagc |
| NC2.9 M13 rev | ttgatgccgttcttctgcttgtacagagaatcgg |
| NC2.9 M14 fwd | gaagaacggcatcaaggtgaactgtttgaagtggc |
| NC2.9 M14 rev | ttgatgccgttcttctgcttgtcttgaccacaac |
| NC2.9 M15 fwd | gaagaacggcatcaaggtgaactcttggtgtggac |
| NC2.9 M15 rev | ttgatgccgttcttctgcttgtatagtttcatgaat |
| NC2.9 M16 fwd | gaagaacggcatcaaggtgaactcattaccccata |
| NC2.9 M16 rev | ttgatgccgttcttctgcttgtccccttgctatg |
| NC2.9 M17 fwd | gaagaacggcatcaaggtgaactcacagatagcaa |
| NC2.9 M17 rev | ttgatgccgttcttctgcttgttccagaggagtc |
| NC2.9 M18 fwd | gaagaacggcatcaaggtgaactgctaccttcagc |
| NC2.9 M18 rev | ttgatgccgttcttctgcttgtcagcactctcct |
| NC2.9 M19 fwd | gaagaacggcatcaaggtgaacttctgtgtcagtc |
| NC2.9 M19 rev | ttgatgccgttcttctgcttgtggtggtaggctt |
| NC2.9 M20 fwd | gaagaacggcatcaaggtgaactctgaccaggata |
| NC2.9 M20 rev | ttgatgccgttcttctgcttgtgaagcttttgatg |
| NC2.9 M21 fwd | gaagaacggcatcaaggtgaacttaagtgttgtattc |
| NC2.9 M21 rev | ttgatgccgttcttctgcttgtaggcagccactg |
