# Supplementary material for: Dynamic and Differential Regulation of Stem Cell Factor FoxD3 in the Neural Crest Is Encrypted in the Genome
Source: PLoS Genet. 2012 Dec 20;8(12):e1003142. doi: 10.1371/journal.pgen.1003142 (PMC3527204; doi:10.1371/journal.pgen.1003142)
Supplement: Table S3 — Primers used for binding site mutations of NC1. Text in capitals indicates mutated sequence. To make the mutated constructs, primers were paired with flanking primers NC1.1, amplified and joined in a fusion PCR reaction using the flanking NC1.1 primers. (DOCX) [file pgen.1003142.s006.docx]

**Supplemental Table 3.** **Primers used for binding site mutations of NC1.**

| **Primer name** | **Primer sequence** |
| --- | --- |
| NC1.1 Ikaros mut. fwd | gactacaagagccctattctccta |
| NC1.1 Ikaros mut. rev | agggctcttgtagtctaatgagctt |
| NC1.1 Ets/Zeb mut. fwd | ctatttagaacagtaattagatttta |
| NC1.1 Ets/Zeb mut. rev | tactgttctaaataggccaggga |
| NC1.1 HD mut. fwd | cctactaccaccgattttaacagg |
| NC1.1 HD mut. rev | atcggtggtagtaggagaatagg |
| NC1.1 Ets/Gata mut. fwd | ttaaacaacagctcaacagatcag |
| NC1.1 Ets/Gata mut. rev | tgagctgttgtttaaaatctaattac |
